# Single-cell transcriptomic analysis reveals fatty acid metabolic heterogeneity and identifies DKK1 as a candidate prognostic factor in osteosarcoma

**DOI:** 10.3389/fendo.2026.1918915

**Published:** 2026-09-07

**Authors:** Lifang Wang, Meiyan Gao, Zhizhong Liang

**Affiliations:** 1 Department of Comprehensive Medicine, Shanxi Province Cancer Hospital, Shanxi Hospital Affiliated to Cancer Hospital, Chinese Academy of Medical Sciences, Cancer Hospital Affiliated to Shanxi Medical University, Taiyuan, China; 2 Department of Bone and Soft Tissue Oncology, Shanxi Province Cancer Hospital, Shanxi Hospital Affiliated to Cancer Hospital, Chinese Academy of Medical Sciences, Cancer Hospital Affiliated to Shanxi Medical University, Taiyuan, China

**Keywords:** DKK1, fatty acid degradation, osteosarcoma, single-cell transcriptomics, tumor microenvironment

## Abstract

Osteosarcoma (OS) is an aggressive primary bone malignancy that predominantly affects children and adolescents. Although surgery and chemotherapy have improved survival, therapeutic progress has remained limited over recent decades. Aberrant fatty acid metabolism has been increasingly implicated in OS progression, yet its distribution across different cellular compartments of the tumor microenvironment remains unclear. In this study, we integrated single-cell RNA-sequencing data from 140,562 cells across 17 OS patients and characterized the metabolic heterogeneity of tumor microenvironment. Two fatty acid metabolism-associated populations were identified: IBSP+ malignant osteosarcoma cells and SPARC+ macrophages. IBSP+ malignant cells showed preferential activation of fatty acid degradation and occupied an intermediate state along the pseudo-time trajectory. Lipid uptake and cholesterol efflux programs reached their highest activity near this state, indicating substantial lipid metabolic remodeling along the inferred transition among malignant-cell states. By contrast, SPARC^+^ macrophages exhibited a lipid-adapted, immunosuppressive phenotype accompanied by elevated fatty acid degradation activity. Cell–cell communication analysis further identified IBSP^+^ malignant cells as a major source of APOE- and SPP1-related signals directed toward SPARC+ macrophages, suggesting coordinated metabolic communication between these populations. To evaluate its clinical relevance, we applied transcriptomic deconvolution to independent OS cohorts. Concurrent enrichment of IBSP+ malignant cells and SPARC+ macrophages was associated with significantly shorter overall survival. Comparison of the double-high and double-low groups subsequently identified DKK1 as a candidate effector linked to this adverse metabolic niche. Consistent with this observation, DKK1 silencing in MG-63 cells reduced proliferation, colony formation, migration, and invasion. Collectively, these findings characterize fatty acid metabolism-associated cellular heterogeneity and a clinically relevant association between IBSP^+^ malignant cells and SPARC^+^ macrophages in OS, while identifying DKK1 as a candidate prognostic and therapeutic factor associated with the double-high phenotype.

## 1 Materials and methods

### 1.1 Data collection and integration

The osteosarcoma scRNA-seq data were obtained from the Gene Expression Omnibus (GEO) database, including GSE152048 and GSE162454 (1). Meanwhile, bulk RNA-sequencing datasets and corresponding clinical information were obtained from the TARGET-OS project and the GEO datasets GSE21257 and GSE33382.

### 1.2 scRNA-seq quality control, batch correction and data processing

The scRNA-seq data analyses were performed using the Seurat (v4.2) (2–4). Cells expressing fewer than 500 genes, or more than 6,000 genes were excluded. Cells with mitochondrial transcript proportions greater than 15% were also removed. After quality control, gene expression data were normalized using the “NormalizeData” function. The top 2,000 highly variable genes were identified using the “FindVariableFeatures” function with the “vst” method. The data integration was performed using the Harmony package.

### 1.3 Cell clustering and annotation

Graph-based clustering was performed using the “FindNeighbors” and “FindClusters” functions. The clustering resolution was adjusted between res 0.5. Cell types were annotated based on canonical marker genes. The malignant cells and macrophages were separately extracted and were separately subsetted and re-clustered to identify functionally distinct subpopulations.

### 1.4 Copy number variation analysis

To distinguish malignant cells from non-malignant populations, copy number variation (CNV) analysis was performed using the inferCNV package. B cells served as the normal reference population. Large-scale chromosomal gains and losses were inferred from gene expression patterns across genomic regions. The CNV score of each cell was calculated as the sum of the squared inferred CNV values across all genomic regions to quantify genomic instability among malignant cell subsets.

### 1.5 Functional enrichment and metabolic analysis

Gene Set Variation Analysis (GSVA) and single-sample Gene Set Enrichment Analysis (ssGSEA) were conducted using the GSVA package (5, 6). Hallmark gene sets and metabolic pathways were downloaded from the Molecular Signatures Database (MSigDB) and Kyoto Encyclopedia of Genes and Genomes (KEGG) (7–9). The fatty acid degradation gene set (KEGG hsa00071) contained 42 genes, as listed in **Supplementary Table 1**. GSVA was performed with method = “gsva”, kcdf = “Gaussian”, min.sz = 1, max.sz = 500, and mx.diff = TRUE; all other parameters were set to default. Pathway scores were subsequently standardized by z-score normalization. Correlations between cell abundance and metabolic pathway activity were evaluated using Pearson’s or Spearman’s correlation analysis, as appropriate.

### 1.6 pseudo-time trajectory analysis

Cell differentiation potential was estimated using “CytoTRACE2”, which infers developmental states based on transcriptional diversity (10, 11). To reconstruct dynamic developmental trajectories, pseudotime analysis was performed using Monocle2 (10, 12). Differentially expressed genes associated with pseudo-time progression were identified using the differentialGeneTest function with adjusted qvalue < 1×10^-5^.

### 1.7 Cell-cell communication analysis

Intercellular communication networks were inferred using “CellChat” (v2.0) (13). Known ligand-receptor interactions were used to quantify communication probability and interaction strength among different cell populations. Incoming and outgoing signaling patterns were analyzed to identify dominant signaling pathways involved in tumor-macrophage interactions.

### 1.8 Bulk RNA-seq dataset deconvolution and survival analysis

The scRNA-seq-derived gene signatures were used to deconvolute bulk RNA-seq datasets. Relative infiltration scores of specific cellular populations, including IBSP+ malignant cells and SPARC+ macrophages, were estimated using “CIBERSORTx” (14, 15). A CIBERSORTx signature matrix was generated from the annotated single-cell RNA-sequencing dataset. Population-specific marker genes were identified using the Seurat FindAllMarkers function with the Wilcoxon rank-sum test. Genes with an adjusted *P* value < 0.05, an average log_2_ fold change > 0.25, and expression in at least 25% of cells in the target population were retained, whereas genes lacking population specificity were excluded. CIBERSORTx was run in relative mode with 1,000 permutations, S-mode batch correction, and quantile normalization disabled. Only samples with a deconvolution *P* value < 0.05 were included in subsequent analyses. Patients were stratified into high- and low-infiltration groups according to optimal cutoff values. Kaplan-Meier survival curves were generated using the survival and “survminer” packages in R. Differences in overall survival were assessed using the log-rank test.

### 1.9 Identification and prioritization of DKK1

DKK1 was identified through a stepwise screening strategy. Differentially expressed genes in IBSP^+^ malignant cells with high fatty acid degradation activity were first identified using an adjusted P value < 0.05 and |log_2_ fold change| > 0.25. CellChat was then used to predict ligand–receptor interactions between IBSP^+^ malignant cells and SPARC^+^ macrophages. Secreted ligands associated with fatty acid degradation scores and with corresponding receptors expressed in SPARC^+^ macrophages were prioritized. Their clinical associations with macrophage infiltration and prognosis were subsequently evaluated in the TARGET-OS cohort, leading to the identification of DKK1 as a candidate secreted factor.

### 1.10 Cell culture and siRNA transfection

The cell lines MG-63, U2OS, HOS, and Saos-2 were obtained from the American Type Culture Collection (ATCC). Cells were cultured in Dulbecco’s Modified Eagle Medium (DMEM) supplemented with 10% fetal bovine serum and 1% penicillin-streptomycin. Cultures were maintained at 37 °C in a humidified incubator containing 5% CO2. Small interfering RNAs targeting DKK1, and corresponding negative control oligonucleotides were synthesized commercially. Cells were transfected using Lipofectamine 3000 according to the manufacturer’s instructions when reaching 60–80% confluence.

### 1.11 Quantitative real-time PCR

Total RNA was isolated using TRIzol reagent. Complementary DNA was synthesized using a reverse transcription kit according to the manufacturer’s instructions. Quantitative real-time PCR was performed using SYBR Green Master Mix on a real-time PCR detection system (16). Relative gene expression levels were calculated using the 2^-ΔΔCt method, with GAPDH serving as the internal control. The primer sequences for human DKK1 were:

DKK1-F: 5′-GGTATTCCAGAAGAACCACCTTG-3′.
DKK1-R: 5′-CTTGGACCAGAAGTGTCTAGCAC-3′.

### 1.12 Cell proliferation assay

Cell proliferation was evaluated using the Cell Counting Kit-8 (CCK-8) assay. Transfected MG-63 cells were seeded into 96-well plates at a density of 2×10^3^ cells per well. Absorbance at 450 nm was measured at 0–48 hours after incubation with CCK-8 reagent.

### 1.13 Colony formation assay

Transfected cells were seeded into 6-well plates at a density of 1×10^3^ cells per well and cultured for 10–14 days. Colonies were fixed with 4% paraformaldehyde and stained using 0.1% crystal violet. Colonies containing more than 50 cells were counted under a microscope.

### 1.14 Wound healing assay

Cells were seeded in 6-well plates and cultured until reaching complete confluence. A sterile 200 μL pipette tip was used to generate a linear scratch. Detached cells were removed by washing with phosphate-buffered saline. Images were captured at 0 and 48 hours, and migration ability was quantified by measuring wound closure.

### 1.15 Transwell migration and invasion assays

Cell migration and invasion assays were performed using Transwell chambers with 8 μm pore membranes. For invasion assays, the upper chamber membrane was pre-coated with Matrigel. A total of 5 × 10^4^ cells suspended in serum-free medium were added to the upper chamber, while medium containing 20% fetal bovine serum was placed in the lower chamber. After 24 hours of incubation, migrated or invaded cells on the lower membrane surface were fixed, stained with crystal violet, and counted under a light microscope.

### 1.16 Statistical analysis

All statistical analyses were performed using R software (version 4.3.0) and GraphPad Prism. Experimental data are presented as mean ± standard deviation from at least three independent biological replicates.

## 2 Introduction

Osteosarcoma (OS) is the most common primary bone malignancy in children and adolescents and is prone to local invasion and early lung metastasis. Although surgery and chemotherapy have improved survival, therapeutic progress has plateaued in recent decades (17). Although the combination of surgical resection and intensive chemotherapy has improved outcomes for patients with localized disease, the prognosis of patients with recurrent or metastatic osteosarcoma remains poor and has changed little over recent decades (18, 19). Increasing evidence indicates that the OS tumor microenvironment contributes to tumor growth, metastasis, and therapeutic resistance. Therefore, defining the cellular interactions and molecular programs that re-shape the TME is essential for identifying new therapeutic vulnerabilities in OS.

Metabolic reprogramming is an important mechanism by which malignant cells reshape the TME (20). Rapidly growing bone tumors utilize altered nutrient pathways-particularly fatty acid metabolism-to fuel proliferation, metastasis, and chemoresistance (21, 22). These lipid-driven processes likely exacerbate immune suppression by promoting pro-tumor macrophage polarization. However, the specific osteosarcoma cell populations that drive lipid metabolic reprogramming, and how they communicate with macrophage subsets in theTME, remain insufficiently defined.

Tumor-associated macrophages are abundant immune components in osteosarcoma and contribute to angiogenesis, extracellular matrix remodeling, immune suppression, and metastasis (1, 23). Their tumor-promoting states, including M2-like phenotypes, are often linked to aggressive disease and poor prognosis (24, 25). but macrophage polarization is strongly shaped by malignant cell-derived signals. Metabolic reprogramming may be a key mechanism underlying this interaction. In particular, fatty acid metabolism supports osteosarcoma survival, metastasis, and treatment resistance, while lipid accumulation and fatty acid catabolism can drive immunosuppressive macrophage polarization (26, 27). Beyond supporting tumor growth, altered metabolism can reshape the microenvironment through nutrient competition, metabolite exchange, and metabolism-related signaling (28, 29). These findings raise the critical question of whether specific lipid-metabolic osteosarcoma states establish niches that educate macrophages to drive disease progression.

Single-cell RNA sequencing enables systematic dissection of malignant cell states, immune heterogeneity, metabolic programs, and intercellular communication within the same tumor ecosystem (30–33). Although previous studies have described cellular diversity in osteosarcoma, the connection between lipid-metabolic malignant subpopulations and macrophage functional states remains poorly defined. In particular, it is unclear which malignant cell subsets display lipid metabolic activation, whether they preferentially communicate with specific macrophage populations, and whether such interactions are clinically relevant (34).

In this study, we integrated the scRNA-seq from 140,562 cells across 17 osteosarcoma patients and uncovered a fatty acid metabolism-associated interaction between IBSP^+^ malignant cells and SPARC+ macrophages. This cellular axis links malignant-cell lipid remodeling to the formation of an immunosuppressive myeloid microenvironment and unfavorable clinical outcomes. These findings extend the biological significance of fatty acid metabolism beyond tumor-intrinsic growth and highlight metabolic communication between malignant cells and macrophages as an important mechanism underlying osteosarcoma progression. Moreover, the identification and functional validation of DKK1 as a candidate effector provides a potential therapeutic entry point for disrupting this adverse lipid-metabolic niche. Collectively, our study provides new insights into how metabolic heterogeneity shapes the osteosarcoma immune microenvironment and supports the development of metabolism-informed therapeutic strategies.

## 3 Result

### 3.1 Single-cell transcriptomic profiling reveals the cellular landscape and metabolic heterogeneity of OS

**Figure 1:**
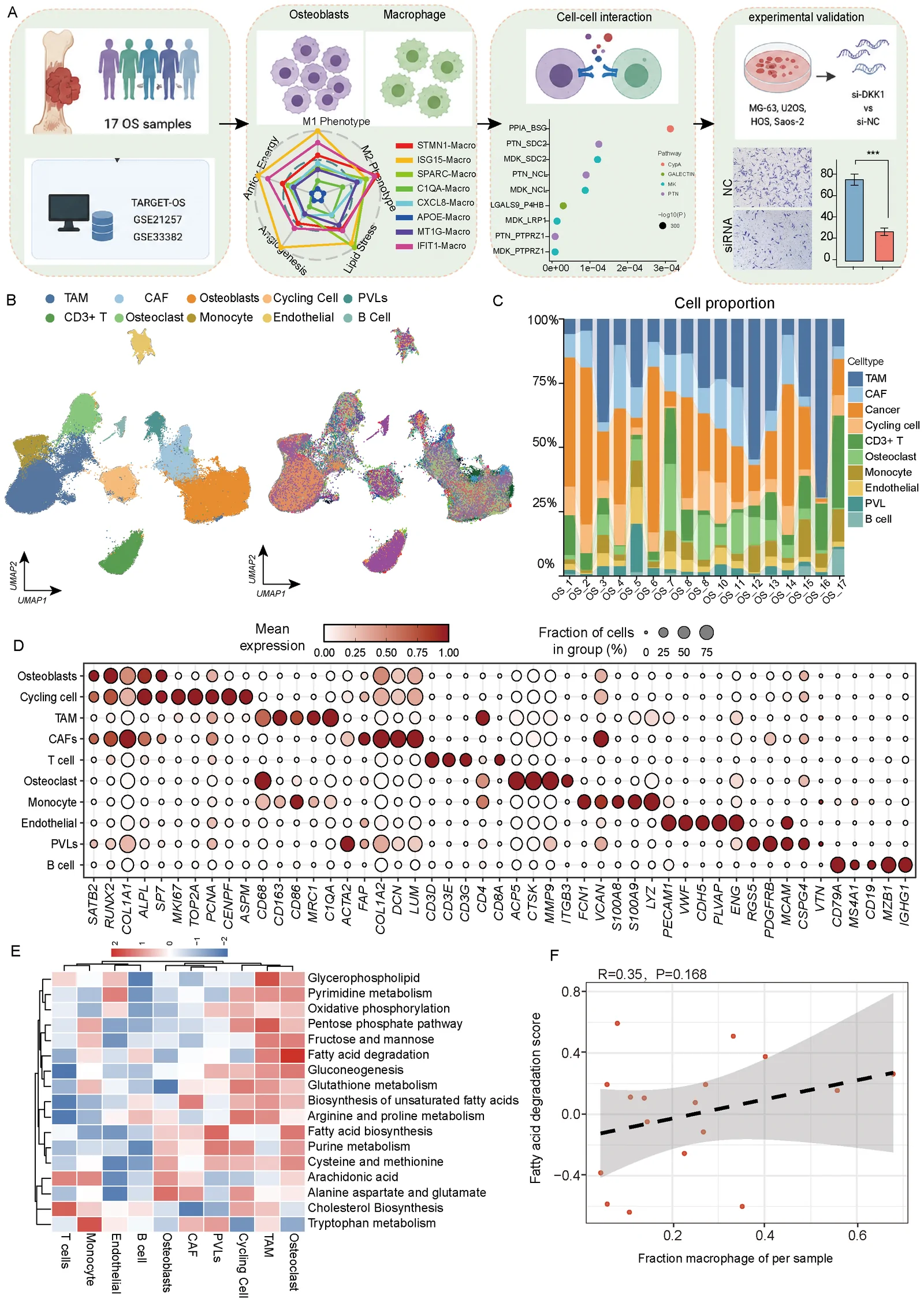
The single-cell transcriptomic landscape and metabolic heterogeneity of human osteosarcoma. **(A)** Workflow illustrating the collection, integration, and analysis of the osteosarcoma single-cell RNA sequencing dataset. **(B)** UMAP plots of 140,562 individual cells colored by identified major cell types (left) and sample origins (right), demonstrating the cellular atlas of the osteosarcoma microenvironment. **(C)** Bar plot showing the relative proportions and distribution of the 10 major cell lineages across 17 individual osteosarcoma patient samples. **(D)** Bubble plot displaying the mean expression levels (color intensity) and the percentage of expressing cells (circle size) of lineage-specific canonical marker genes used for cell-type identification. **(E)** Heatmap demonstrating the enrichment scores of representative metabolic pathways across different cell lineages, calculated via GSVA. **(F)** Scatter plot showing the positive correlation between the fraction of TAMs per sample and the fatty acid degradation pathway score (Y-axis), with the dashed line representing the linear regression fit and the gray area indicating the 95% confidence interval.

To systematically elucidate the cellular composition and metabolic architecture of the osteosarcoma (OS) microenvironment, we integrated and analyzed scRNA-seq dataset comprising 140,562 high-quality cells across 17 OS samples as illustrated in the study workflow (**Figure 1A**). Following batch-effect correction via Harmony and unsupervised clustering identified 10 major cell lineages based on canonical marker gene expression (**Figure 1B**). The relative proportion of these cell types exhibited substantial variation across individual patients, highlighting a highly heterogeneous inter-patient cellular ecosystem (**Figure 1C**). These cell lineages encompassed the malignant and stromal compartments, including Cycling cells, Osteoblasts (*SATB2*, *RUNX2*), and cancer-associated fibroblasts (CAFs, *FAP*, *COL1A2*), endothelial cells (*PECAM1*, *VWF*), perivascular-like cells (PVLs, *RGS5*, *PDGFRB*), CD3+T cells (*CD3D*, *CD3E*), B cells (*CD79A*, *MS4A1*), monocytes (*FCN1*, *VCAN*), Osteoclasts (ACP5, CTSK), and Tumor-Associated Macrophages (TAMs, *CD68*, *CD163*) (**Figure 1D**). Given that metabolic reprogramming within the TME profoundly shapes OS progression and immune evasion, we performed metabolic pathway enrichment analysis using GSVA to map the metabolic profiles across these distinct lineages. Distinct metabolic programs were observed across cell populations. Notably, fatty acid degradation emerged as one of the most prominently enriched metabolic pathways, with particularly high activity in TAMs and osteoclasts (**Figure 1E**). To further explore the potential crosstalk between TAM infiltration and these metabolic alterations, we evaluated the correlation between the abundance of TAMs and metabolic pathway scores across the clinical samples. Correlation analysis revealed that the fatty acid degradation score was positively correlated with the fraction of TAMs per sample (R = 0.35, **Figure 1F**), strongly suggesting that a lipid-metabolism-enriched microenvironment may support or be shaped by TAM polarization and accumulation, thereby serving as a potential driver of immunosuppression in OS.

### 3.2 Intratumoral heterogeneity and metabolic reprogramming of malignant osteosarcoma cells

**Figure 2:**
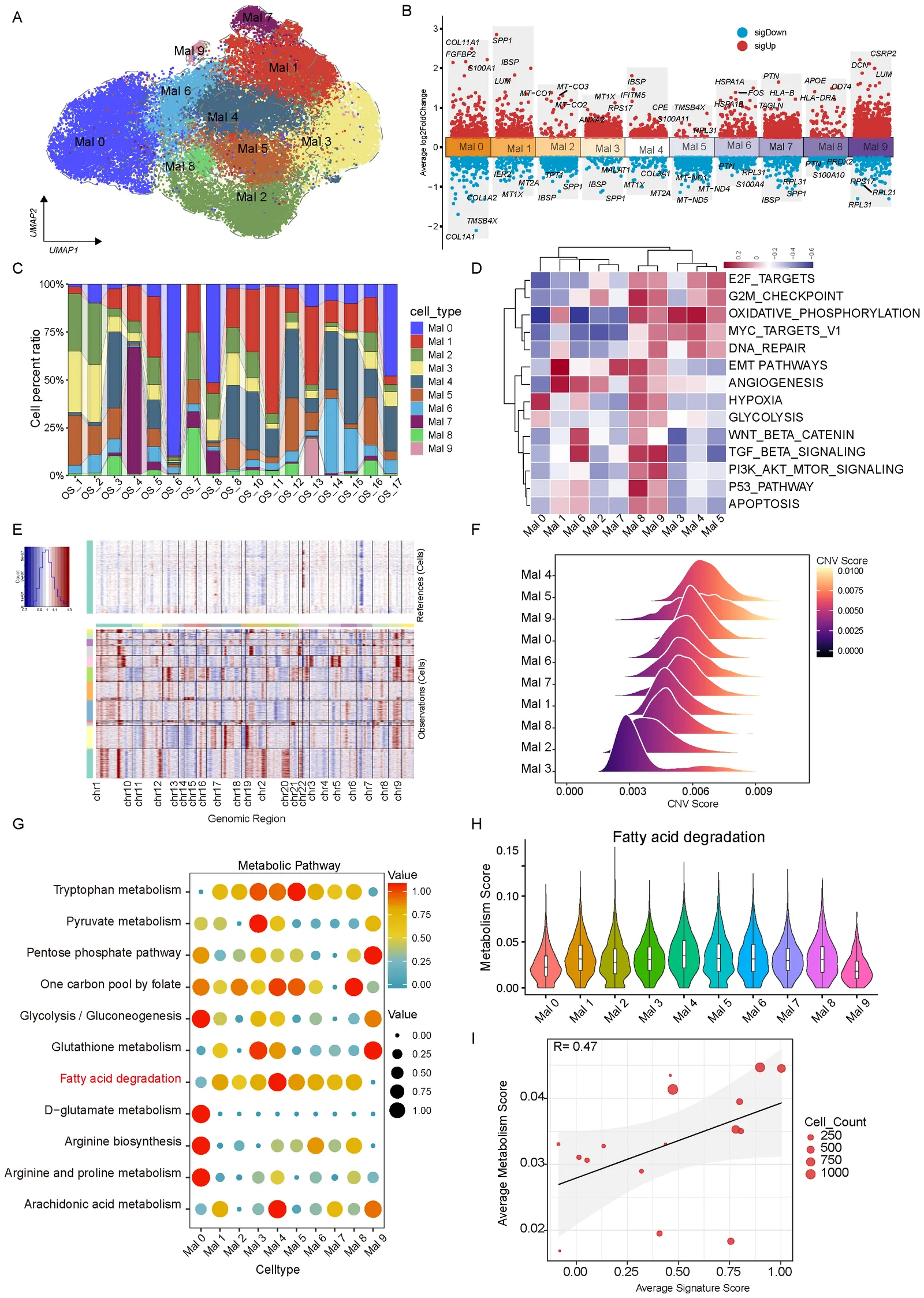
Dissection of transcriptomic heterogeneity, copy number variations, and metabolic profiles in malignant osteosarcoma subclusters. **(A)** UMAP plot of 40,188 malignant osteosarcoma cells, color-coded into 10 distinct subclusters. **(B)** Manhattan-style dot plot highlighting significantly up-regulated (red) and down-regulated (blue) differentially expressed genes (DEGs) across different malignant lineages. **(C)** Percentage bar plot illustrating the relative distribution and composition of the 10 malignant subclusters across the 17 individual patient samples. **(D)** Heatmap of GSVA scores showing the enrichment of representative MSigDB Hallmark pathways (e.g., cell cycle, EMT, and hypoxia) across the malignant subclusters. **(E)** InferCNV heatmap showing large-scale chromosomal amplification (red) and deletion (blue) profiles in observed osteosarcoma cells relative to normal reference cells. **(F)** Ridgeline plot quantifying the global copy number variation (CNV) scores across different malignant subclusters to illustrate genomic instability. **(G)** Bubble plot outlines the relative enrichment values and cell fraction percentages of key metabolic pathways, highlighting the distinct activation of “Fatty acid degradation” in specific clusters like Mal 3 and IBSP+ malignant cells. **(H)** Violin plot showing the distribution of the fatty acid degradation metabolism score across the 10 malignant subclusters. **(I)** Linear regression analysis demonstrates the significant positive correlation (R = 0.47, p = 0.042) between the average fatty acid degradation metabolism score and the tumor signature score, with bubble sizes indicating cell counts.

To further dissect the extensive heterogeneity within the malignant compartment of OS, we extracted and re-clustered 40,188 tumor cells, which re-clustered 10 distinct subclusters (Mal 0 to Mal 9) with specialized transcriptomic profiles (**Figure 2A**). Distinct patterns of differentially expressed genes (DEGs) were identified across these clusters, with prominent up-regulation or down-regulation of classical skeletal and matrix markers (e.g., *COL11A1*, *SPP1*, *IBSP*, and *LUM*), signifying pronounced phenotypic divergence (**Figure 2B**). The relative proportion of these 10 clusters varied dynamically across the 17 individual OS samples, indicating extensive inter-patient and intratumoral cellular architecture heterogeneity (**Figure 2C**). GSVA of the Hallmark gene sets revealed distinct functional programs across the malignant subclusters. Mal 0 and Mal 8 were enriched in cell-cycle-related pathways, including E2F targets and the G2M checkpoint, whereas Mal 1 showed strong activation of EMT and angiogenesis. WNT/β-catenin and TGF-β signaling were more active in Mal 6, while IBSP+ malignant cells (IBSP) was characterized by increased oxidative phosphorylation and MYC target activity, together with relatively active proliferative programs. These results demonstrate the functional heterogeneity of malignant cells and suggest that IBSP+ malignant cells high expressed IBSP represents a metabolically active tumor-cell state (**Figure 2D**). To validate the malignant nature of these clusters, we used inferCNV to estimate large-scale chromosomal copy number alterations. Widespread CNV alterations were observed across the malignant subclusters compared with normal reference cells (**Figure 2E**). Notably, IBSP+ malignant cells exhibited the highest overall CNV score among the subclusters, further supporting its pronounced genomic instability and malignant characteristics (**Figure 2F**). Crucially, given the systemic lipid alterations observed in the broader ecosystem, we profiled the metabolic landscape of these malignant subclusters (**Figure 2G**). Distinct subclusters demonstrated partitioned metabolic preferences; notably, cluster IBSP+ malignant cells exhibited a robust and preferential activation of the fatty acid degradation pathway (**Figure 2H**). Further correlation analysis confirmed a strong, statistically significant positive correlation between the average metabolic score of fatty acid degradation and the malignant signature score across these tumor cells (R = 0.47, p = 0.042; **Figure 2I**), suggesting that heightened lipid catabolism is intrinsically linked to the malignant property and metabolic adaptation of specific OS cell states.

### 3.3 Deciphering the differentiation trajectory and dynamic metabolic evolution of malignant osteosarcoma cells

**Figure 3:**
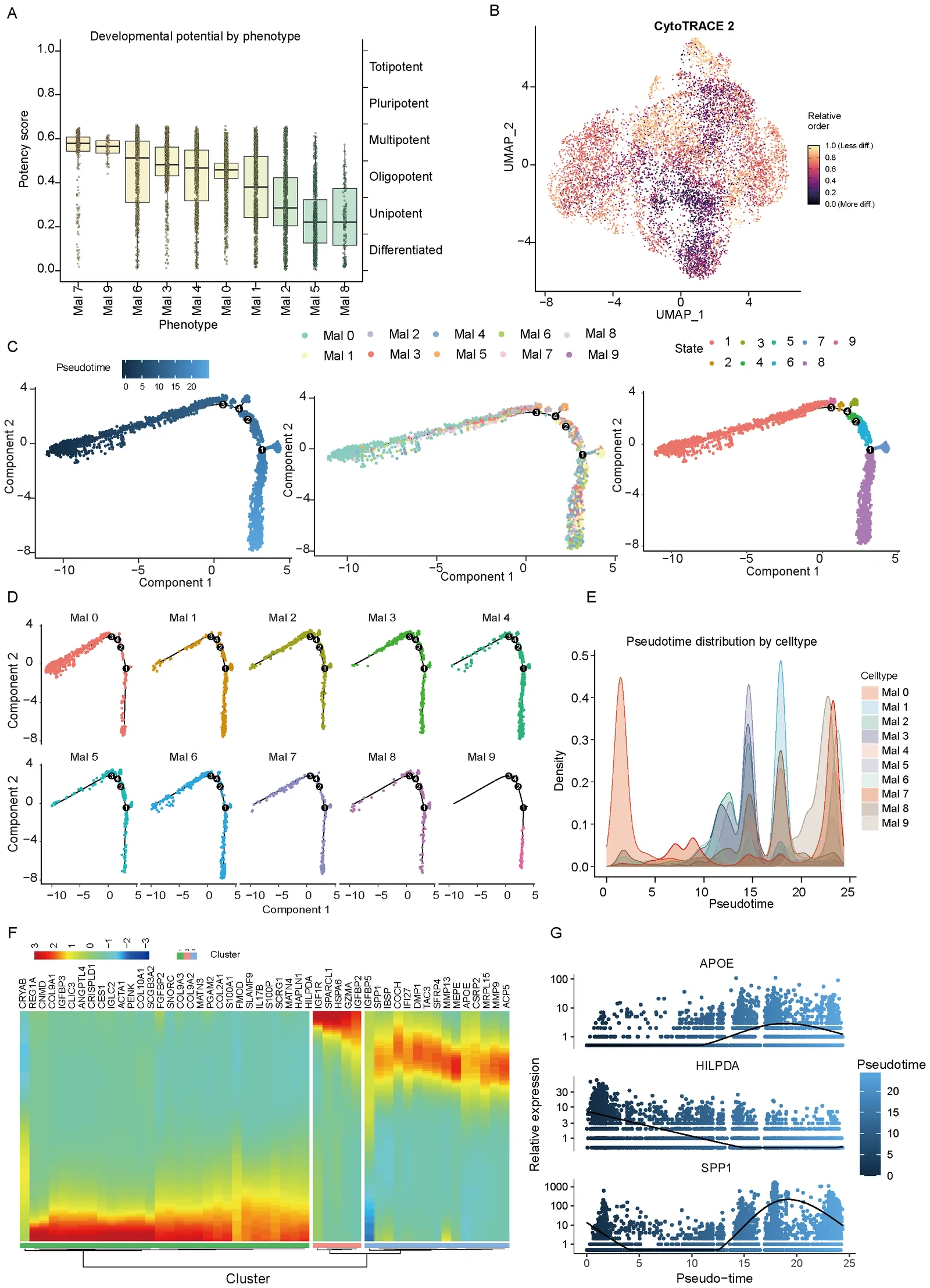
Pseudo-time trajectory and dynamic expression patterns of lipid-metabolism-related genes along the differentiation axis of osteosarcoma cells. **(A)** Box plot quantifying the developmental potency scores of the 10 malignant osteosarcoma subclusters (Mal 0 to Mal 9) predicted by CytoTRACE 2, ranging from differentiated to totipotent statuses. **(B)** UMAP plot colored by CytoTRACE 2 relative differentiation order, showing the spatial gradient of cellular differentiation potential. **(C)** Monocle 2 pseudotime trajectory plots of malignant osteosarcoma cells colored by pseudotime gradient (left), cell subclusters (middle), and trajectory states (right). **(D)** Individual trajectory plots showcasing the specific distribution and alignment of each malignant subcluster (Mal 0 to Mal 9) along the continuous pseudotime timeline. **(E)** Density plot showing the relative distribution and enrichment frequency of different malignant cell subclusters across the pseudotime axis. **(F)** Hierarchical clustering heatmap displaying the dynamic expression profiles of top pseudotime-dependent genes grouped into distinct temporal expression modules. **(G)** Kinetic expression scatter plots depicting the continuous relative expression levels of key lipid-metabolism and microenvironment-modulating genes (APOE, HILPDA, and SPP1) across the pseudotime axis, with solid black lines representing the smoothed gene expression trends.

To define the developmental relationships among malignant osteosarcoma subclusters, we first assessed their differentiation potential using CytoTRACE 2 (**Figures 3A, B**). Mal 7 and Mal 9 showed higher developmental potency, whereas Mal 5 and Mal 8 displayed more differentiated states. IBSP+ malignant cells exhibited an intermediate potency score, suggesting that it represents a transitional malignant state. Monocle 2 pseudotime analysis further reconstructed a continuous trajectory comprising nine cellular states (**Figure 3C**). The malignant subclusters were distributed differently along this trajectory: Mal 0 was mainly located at the early stage, IBSP+ malignant cells was enriched in the intermediate-to-late stage, and Mal 5 and Mal 6 accumulated near the terminal stage (**Figures 3D, E**). Dynamic gene-expression analysis revealed sequential changes in skeletal development, extracellular matrix organization, and metabolic programs along pseudo-time (**Figure 3F**). Notably, lipid uptake and cholesterol efflux programs reached their highest activity around the transitional stage occupied by IBSP+ malignant cells, indicating substantial lipid metabolic remodeling during malignant-cell differentiation. Consistently, HILPDA expression was highest at the beginning of pseudo-time and gradually declined, whereas the lipid-transport regulator *APOE* increased during the intermediate-to-late stages. *SPP1* was also markedly induced at later stages, suggesting enhanced microenvironment-remodeling activity during malignant progression (**Figure 3G**). Together, these results place IBSP+ malignant cells at a key transitional stage characterized by active lipid metabolic remodeling and suggest that changes in lipid handling are closely linked to the differentiation of malignant osteosarcoma cells.

### 3.4 Identification of a lipid-metabolism-enriched SPARC+TAM subpopulation driving M2 polarization

**Figure 4:**
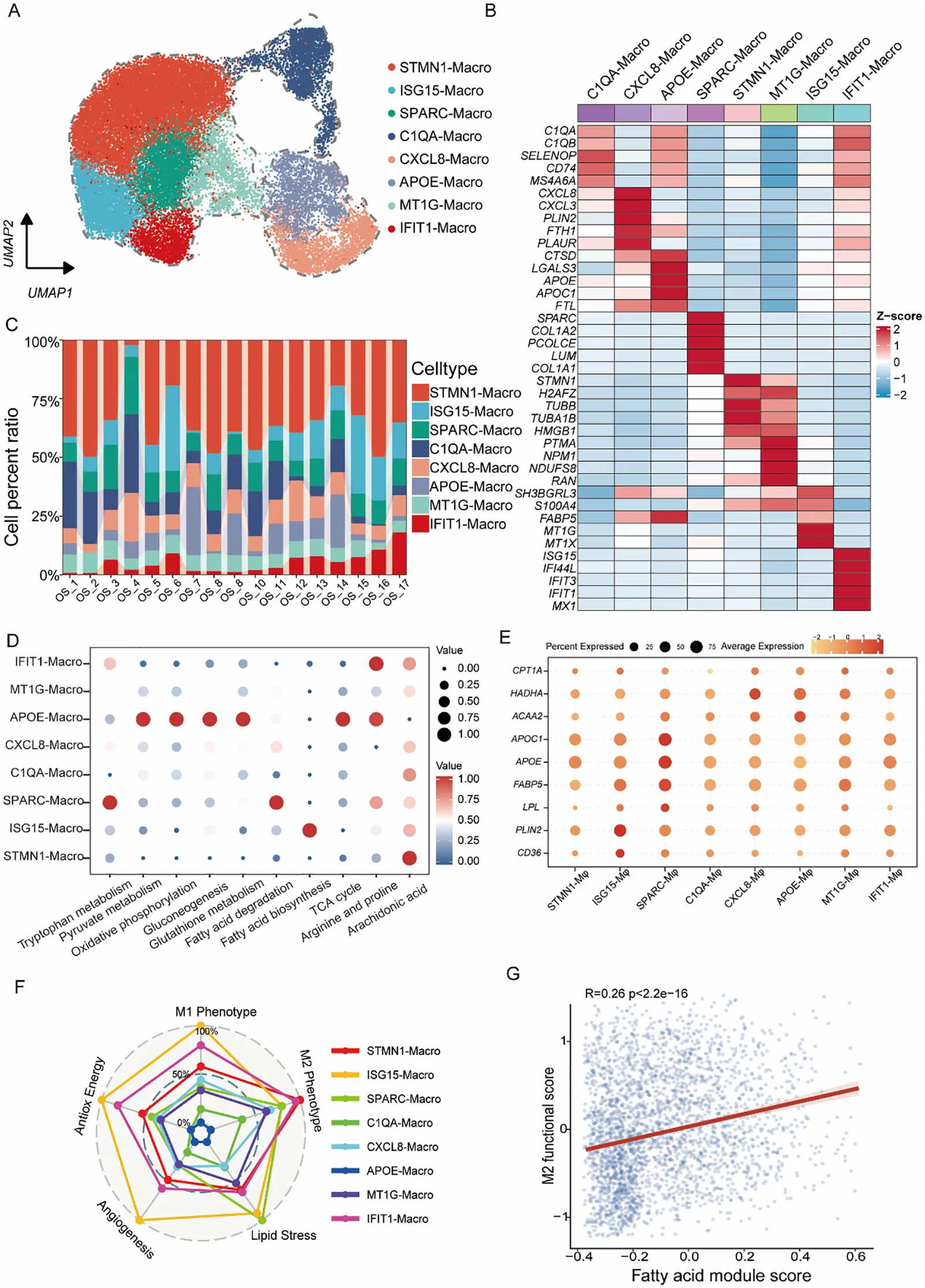
Characterization of TAM subclusters and identification of a SPARC+M2-polarized population tailored to fatty acid degradation. **(A)** UMAP plot displaying the sub-clustering analysis of 32,427 TAMs into eight distinct sub-populations annotated by cell-type markers. **(B)** Graded Z-score heatmap illustrating the expression levels of the top lineage-specific marker genes identifying each TAM subcluster. **(C)** Relative percentage stack bar chart illustrating the proportion and architectural distribution of the eight TAM subclusters across individual patient samples. **(D)** Bubble plot highlighting functional enrichment levels (color scale) and cell fractions (circle size) of prominent carbon and lipid metabolic pathways across the individual TAM lineages, highlighting the high lipid-metabolic state of SPARC+Mφ. **(E)** Dot plot showing the expression of representative lipid-handling genes across macrophage subclusters; dot size indicates the percentage of expressing cells and color indicates average expression. **(F)** Radar plot comparing M1-like, M2-like, lipid-stress, angiogenesis, antioxidant-energy, and related signature scores across macrophage subclusters. **(G)** Scatter plot showing the association between fatty acid module scores and M2-like signature scores across macrophage cells; the analysis should account for patient-level clustering.

To uncover the precise macrophage populations driving the lipid metabolism cross-talk identified in the overall OS ecosystem, we re-clustered 32,427 macrophages (**Figure 4A**). This analysis distinguished eight phenotypically discrete TAM lineages annotated by their top signature genes, including STMN1+Macro, ISG15+Macro, SPARC+Mφ, C1QA+Macro, CXCL8+Macro, APOE+Macro, MT1G+Macro, and IFIT1+Macro, each exhibiting highly coordinated specific gene expression clusters (**Figure 4B**). The compositional landscape of these TAM subclusters showed notable variation across the 17 clinical samples, yet the SPARC+Mφ cluster remained consistently present across multiple patient cohorts (**Figure 4C**). The functional metabolic profiling across these populations revealed that the SPARC+Mφ cluster possessed a uniquely heightened metabolic signature, characterized by intense enrichment in fatty acid degradation, pyruvate metabolism, oxidative phosphorylation, and the TCA cycle (**Figure 4D**). To define the functional polarization profiles of these clusters, we constructed a multi-dimensional radar ecosystem evaluating classical functional states (**Figure 4E**). While clusters like STMN1+Macro mapped to M1-like or angiogenic programs, the SPARC+Mφ subpopulation was defined by prominent M2 phenotype scores and intensive lipid stress reactions (**Figure 4F**). To globally establish this connection within the complete TAM compartment, we correlated the overall fatty acid module scores with empirical M2 functional scores across all single cells. Linear regression validated a highly significant positive correlation (R = 0.26, **Figure 4G**), demonstrating that elevated intrinsic fatty acid catabolism is strongly coupled to the immunosuppressive M2 polarization state of TAMs within the TME. Collectively, SPARC+ macrophages represent a fatty-acid-degradation-enriched, lipid-stressed, and M2-like immunosuppressive TAM population in OS.

### 3.5 Trajectory analysis reveals lipid metabolic reprogramming of SPARC+Mφ in osteosarcoma

**Figure 5:**
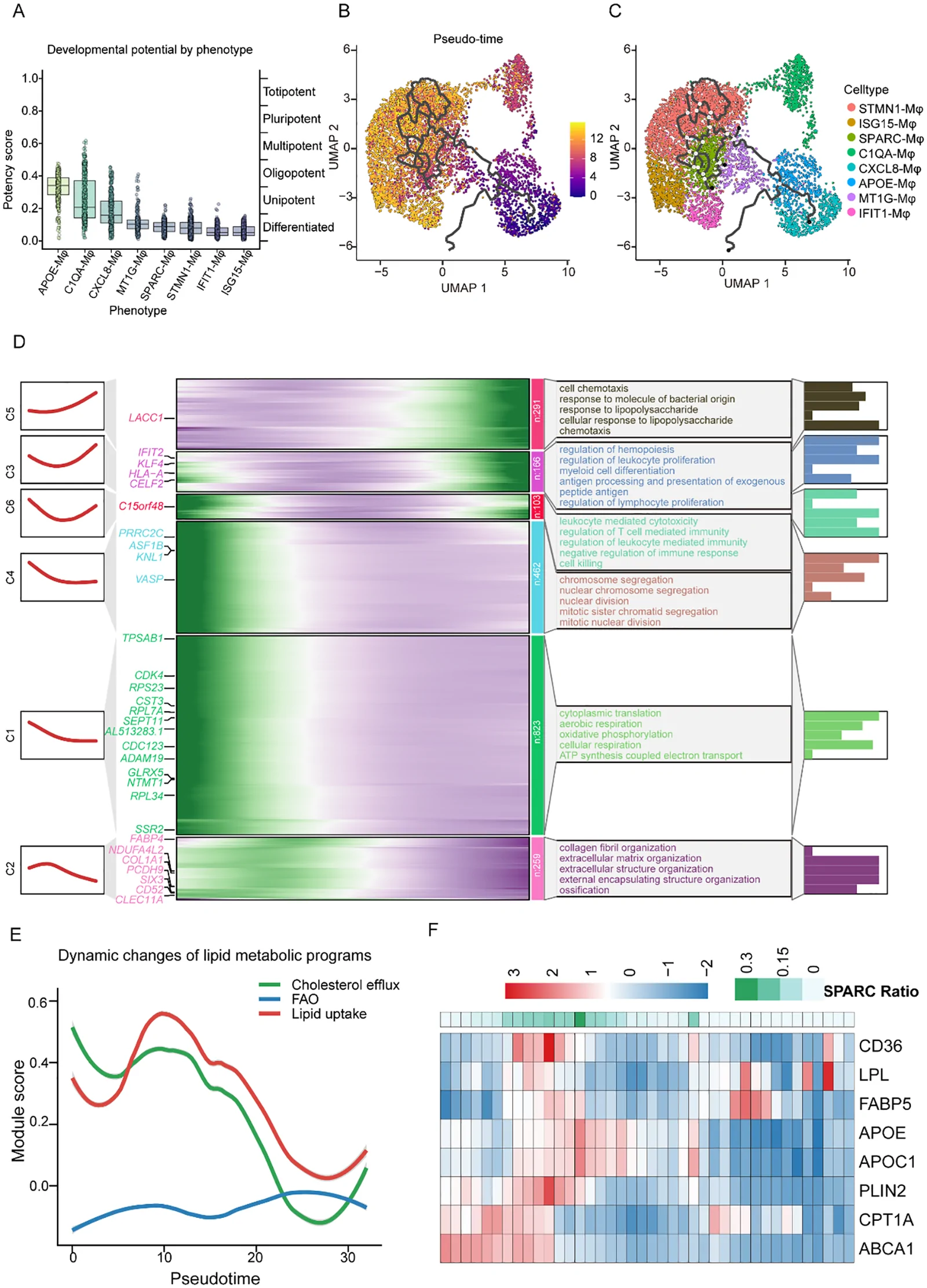
Trajectory analysis reveals lipid metabolic reprogramming of SPARC+Mφ in osteosarcoma. **(A)** Developmental potential analysis of macrophage subsets showing distinct potency states across phenotypes. **(B, C)** Pseudo-time trajectory mapping of osteosarcoma-associated macrophages, displayed by pseudo-time value **(B)** and cell subtype annotation **(C)**, illustrating continuous state transitions during macrophage differentiation within the osteosarcoma microenvironment. **(D)** Heatmap of dynamically expressed genes along pseudo-time, revealing distinct transcriptional modules associated with different developmental states. Representative biological processes enriched in each module are shown on the right. **(E)** Dynamic changes in lipid metabolic programs along pseudotime. Lipid uptake/storage and cholesterol efflux scores increased during the early-to-intermediate stages and declined at later stages. **(F)** Heatmap showing pseudo-time-dependent expression patterns of representative lipid metabolism-related genes, including *CD36*, *LPL*, *FABP5*, *APOE*, *APOC1*, *PLIN2*, *CPT1A*, and *ABCA1*.

To characterize the developmental heterogeneity of osteosarcoma-associated macrophages, we performed trajectory analysis across the macrophage subsets. CytoTRACE 2 revealed differences in differentiation potential, with APOE^+^ Macro and SPARC+Mφ showing relatively high potency scores, whereas ISG15^+^ Macro and IFIT1^+^ Macro exhibited more differentiated states (**Figure 5A**). Pseudo-time analysis further reconstructed a continuous trajectory, indicating progressive changes in macrophage states within the OS microenvironment (**Figures 5B, C**). Genes that varied along pseudo-time were grouped into several temporal expression modules (**Figure 5D**). Early-stage cells were enriched in ribosomal activity, mitochondrial respiration, oxidative phosphorylation, and ATP synthesis, reflecting a metabolically active state. Intermediate stages were characterized by immune-regulatory programs related to hematopoiesis, leukocyte proliferation, T-cell-mediated immunity, and antigen presentation. By contrast, terminal states were enriched in collagen fibril organization, extracellular matrix organization, and ossification, suggesting a shift toward a matrix-remodeling phenotype. Lipid metabolic programs also changed along with the trajectory. Lipid uptake and storage, together with cholesterol efflux, increased from early to intermediate pseudo-time and subsequently declined at later stages, whereas fatty acid oxidation showed relatively modest changes (**Figure 5E**). Consistent with these patterns, CD36, LPL, FABP5, APOE, APOC1, PLIN2, CPT1A, and ABCA1 exhibited clear pseudo-time dependent expression (**Figure 5F**). Genes involved in lipid uptake, transport, and storage were particularly active in trajectory regions enriched in SPARC^+^ Mφ. These findings suggest that SPARC^+^ Mφ represents a lipid-adapted macrophage state characterized by active lipid handling and extracellular matrix remodeling.

### 3.6 Deciphering the cell-cell communication networks associated IBSP+ malignant cells and SPARC+Macrophages crosstalk

**Figure 6:**
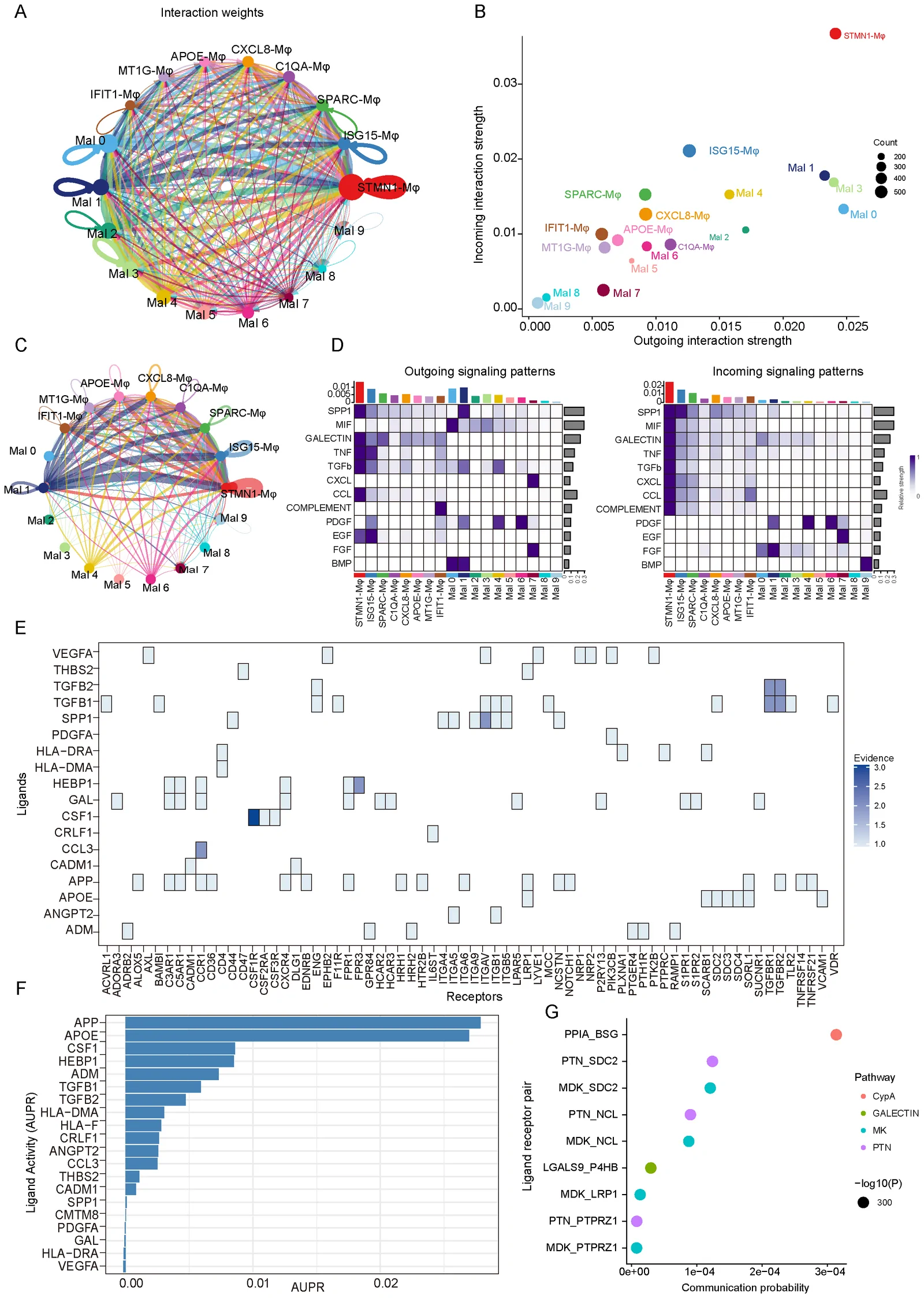
Cell-cell communication landscape revealing the molecular crosstalk between the fatty-acid-degradation-high IBSP+ malignant cells subcluster and TAM lineages. **(A)** Network circle plot displaying the dense interaction weights and molecular communication pathways among malignant cell subclusters (Mal 0 to Mal 9) and TAM lineages. **(B)** Scatter plot comparing the overall outgoing and incoming interaction strengths across all cell populations, highlighting the IBSP+ malignant cells subcluster as a primary signal emitter and SPARC+Mφ as a major signal receiver. **(C)** Network circle plot emphasizing the designated incoming and outgoing cellular interaction pathways centered around the malignant cell network. **(D)** Heatmaps illustrate the prominent outgoing (left) and incoming (right) signaling patterns across the identified lineages, highlighting the tumor-to-macrophage dominance of the SPP1 signaling axis driven by the malignant compartment. **(E)** Dot plot profiling specific ligand-receptor pairs operating between the interacting compartments, with the color intensity representing the supporting experimental evidence count for IBSP+ malignant cells-TAM interactions. **(F)** Bar plot ranking the predicted upstream signaling ligands based on their functional Ligand Activity (AUPR scores), identifying APP, APOE, CSF1, and SPP1 as critical drivers of microenvironmental reprogramming. **(G)** Dot plot showing predicted ligand-receptor interactions between SPARC+ macrophages and the IBSP-high malignant subcluster. Dot size represents -log10(P), and color indicates the annotated signaling pathway; the direction of communication and the method used to calculate communication probability should be specified.

Based on the lipid-metabolic features of IBSP+ malignant cells and SPARC^+^ macrophages, we next used CellChat to investigate the intercellular communication between these two populations (**Figure 6A**). Evaluation of the global interaction weights and strengths revealed intense, dense communication pathways operating between the malignant subclusters and the diverse TAM lineages (**Figure 6B**). Strikingly, by mapping the incoming and outgoing signaling hierarchies, we observed that the fatty-acid-degradation-high IBSP+ malignant cells subcluster, along with Mal 1 and Mal 3, acted as the primary contributors to outgoing signals within the ecosystem, whereas the lipid-stress-dominant SPARC+Mφ subpopulation stood out as a principal receiver for incoming microenvironmental cues (**Figure 6C**). Intriguingly, analysis of targeted signaling patterns showed that the SPP1 signaling axis was heavily driven by outgoing signals derived from these specific malignant cells and was preferentially channeled into incoming receptors on TAM subclusters, notably SPARC+Mφ (**Figure 6D**). To characterize the exact molecular links mediating this metabolic-phenotypic axis, we examined specific ligand-receptor pairs, which revealed significant interactions between IBSP+ malignant cells-derived ligands (SPP1, APOE, TGFB1) and their cognate myeloid receptors, such as the SPP1-CD44 and APOE-SDC4/LRP1 complexes (**Figure 6E**). Furthermore, upstream ligand activity analysis based on AUPR scores independently validated APOE and APP, followed by critical myeloid regulators like CSF1 and SPP1, as the most prominent active functional ligands within this ecosystem (**Figure 6F**). Taken together, these findings provide a cohesive mechanistic loop: the lipid-metabolism-enriched IBSP+ malignant cells actively dispatch key lipid-transport and matrix-remodeling ligands (predominantly APOE and SPP1) to engage specific surface receptors on SPARC+Mφ cells, thereby associated their M2-polarized phenotypic adaptation and maintaining an immunosuppressive metabolic niche in osteosarcoma. To further refine the macrophage-tumor interaction landscape, we focused on the communication between SPARC+Mφ and IBSP+ malignant cells. Dot plot analysis showed that PPIA_BSG exhibited the highest communication probability, followed by PTN_SDC2, MDK_SDC2, PTN_NCL, and MDK_NCL (**Figure 6G**). Additional interactions, including PTN_PTPRZ1, MDK_PTPRZ1, MDK_LRP1, and LGALS9_P4HB, were also detected. These findings suggest that SPARC+Mφ may influence IBSP+ malignant cells mainly through CypA-, PTN-, MDK-, and GALECTIN-related signaling, which are associated with tumor-supportive functions such as cell adhesion, migration, stress adaptation, and microenvironmental remodeling.

### 3.7 Clinical validation of the IBSP+ malignant -SPARC+Mφ axis and identification of DKK1 as a critical promoter of osteosarcoma progression

**Figure 7:**
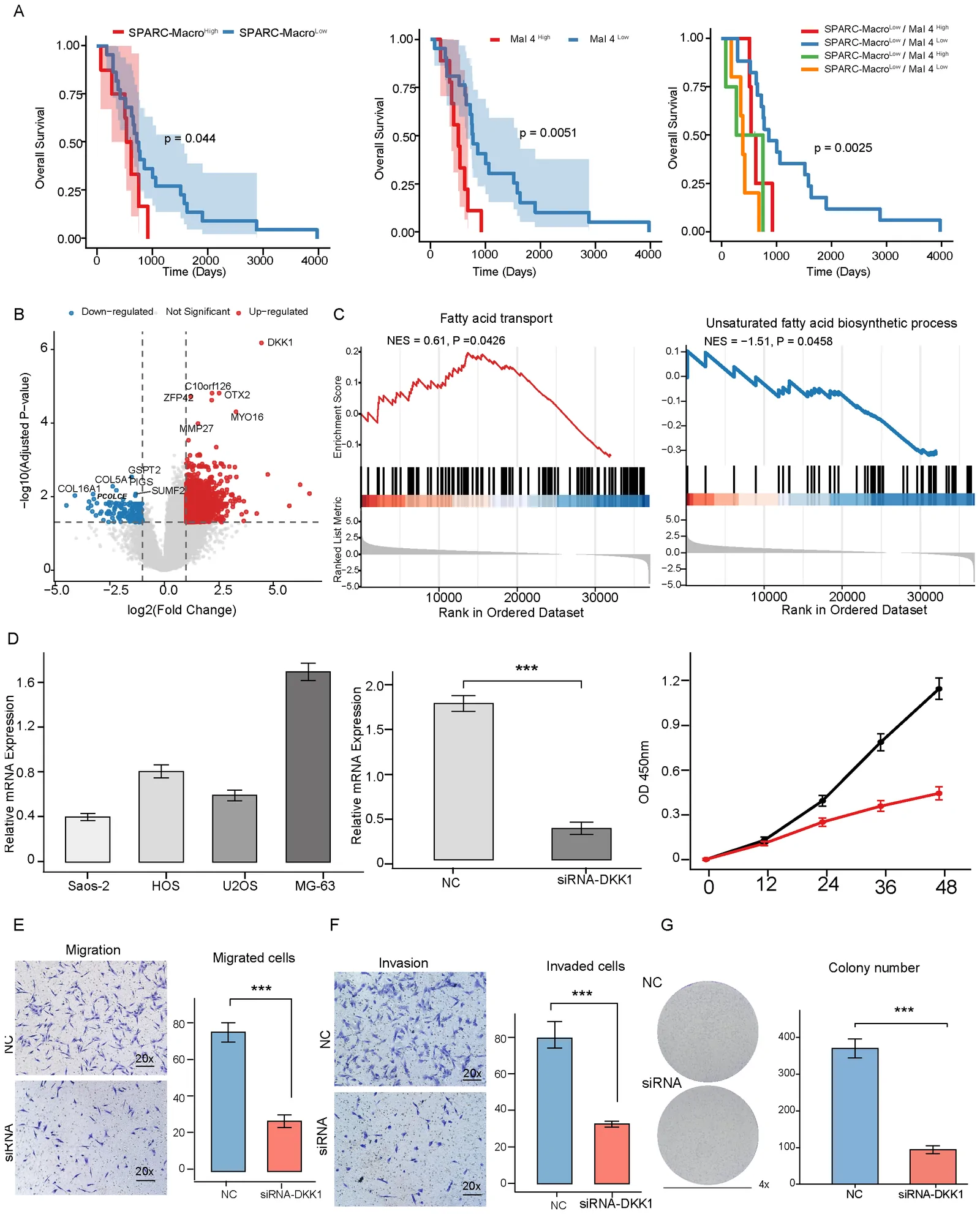
Deconvolution validation of the prognostic axis and *in vitro* characterization of DKK1 in promoting osteosarcoma malignancy. **(A)** Kaplan-Meier overall survival curves of osteosarcoma patients from merged TARGET-OS and GSE33382 cohorts stratified by estimated SPARC+Mφ abundance, IBSP+ malignant cells abundance, and their joint combination groups. **(B)** Volcano plot presenting the differentially expressed genes between the double-high and double-low SPARC+Mφ-IBSP+ malignant cells patient groups, highlighting DKK1 as the most prominent up-regulated gene. **(C)** GSEA enrichment plots showing the significantly altered profiles of Fatty acid transport and Unsaturated fatty acid biosynthetic process pathways between the stratified cohorts. **(D)** Baseline mRNA expression levels of DKK1 across common osteosarcoma cell lines (Saos-2, HOS, U2OS, MG-63) via RT-qPCR (left); verification of siRNA-mediated knockdown efficiency of DKK1 in MG-63 cells (middle); and cell proliferation curves (OD_450) comparing the NC and siRNA-DKK1 groups (right). **(E)** Representative images and statistical quantification of trans well migration assays in MG-63 cells following DKK1 silencing. **(F)** Representative images and quantification of Matrigel invasion assays and crystal violet colony formation assays showing impaired oncogenic capabilities after DKK1 knockdown. **(G)** Representative images and quantification of colony formation in MG-63 cells following DKK1 knockdown. ***P < 0.001.

To assess the clinical relevance of the IBSP+ malignant cell–SPARC+ macrophage axis, we performed transcriptomic deconvolution in independent OS cohorts, including TARGET-OS and GSE33382, to estimate the relative abundance of these two populations. Kaplan-Meier survival analysis revealed that patients with high infiltration scores of either SPARC+Mφ (p = 0.044) or IBSP+ malignant cells (p = 0.0051) exhibited significantly worse overall survival (**Figure 7A**). Stratifying patients based on a combination of both scores demonstrated that the double-high group SPARC+Mφ High IBSP+ malignant cells. High carried the poorest clinical outcomes (p = 0.0025), highlighting a potent synergistic effect of this cellular crosstalk in suggesting osteosarcoma progression (**Figure 7A**). To unveil the molecular underpinnings of this synergistic malignancy, we identified differentially expressed genes (DEGs) between the double-high and double-low patient clusters (**Figure 7B**). Among the top up-regulated candidates, DKK1 stood out as the most significantly elevated gene, alongside a marked enrichment of fatty acid transport pathways (NES = 0.61, p = 0.0426) indicated by GSEA (**Figures 7B, C**). Based on these clinical and bioinformatic insights, we selected DKK1 for downstream functional validation *in vitro*. RT-qPCR verified that DKK1 was highly expressed in human osteosarcoma cell lines, particularly in MG-63 cells (**Figure 7D**). Consequently, we successfully knocked down DKK1 expression in MG-63 cells using a specific small interfering RNA (siRNA-DKK1) (**Figure 7D**). Knockdown of DKK1 substantially suppressed the proliferation rate of MG-63 cells over a 48-hour period and dramatically impaired their colony-forming capability (**Figure 7E**). Consistently, wound healing assays confirmed that siRNA-DKK1 treated cells possessed a significantly lower wound closure rate at 48 hours compared to the negative control (NC) group (**Figure 7G**). Taken together, these data establish DKK1 as a prominent downstream effector driven by the cooperative Mal 4-SPARC+Mφ microenvironment, functioning as an essential driver of cell proliferation, migration, and invasive capacity in OS.

## 4 Discussion

Osteosarcoma (OS) is the most common primary malignant bone tumor in children and adolescents and is characterized by high genomic instability, aggressive progression, and early metastatic potential (35, 36). Despite advances in surgery and multi-agent chemotherapy, the prognosis of patients with recurrent or metastatic disease remains poor. Increasing evidence suggests that metabolic reprogramming is a critical driver of tumor progression and microenvironmental remodeling. In particular, lipid metabolism has emerged as an important regulator of tumor growth, immune evasion, and cellular communication within the tumor microenvironment (37). However, the cellular basis underlying lipid metabolic remodeling in osteosarcoma remains incompletely understood.

In this study, single-cell transcriptomic analysis revealed substantial metabolic heterogeneity within osteosarcoma and identified a malignant subpopulation, termed IBSP+ malignant cells, that was highly enriched in fatty acid degradation pathways. Concurrently, we identified a SPARC^+^ macrophage population characterized by elevated SPARC expression and lipid metabolism-associated and immunosuppressive transcriptional features. Cell-cell communication analysis further demonstrated that IBSP+ malignant cells act as major signaling sources by secreting lipid transport- and matrix remodeling-related ligands, particularly APOE and SPP1, which interact with receptors expressed on SPARC-positive macrophages. These findings suggest potential signaling coordination between the two populations. However, they do not directly demonstrate ligand secretion, receptor activation, intercellular lipid exchange, or causal metabolic reprogramming of macrophages.

To evaluate the clinical relevance of these observations, we integrated single-cell signatures with bulk transcriptomic cohorts. Patients with simultaneous enrichment of both IBSP+ malignant cells and SPARC-positive macrophages exhibited significantly worse overall survival, indicating that cooperation between these two cell populations contributes to disease aggressiveness. Comparative analysis between high-risk and low-risk groups identified DKK1 as one of the most significantly upregulated genes in the aggressive osteosarcoma niche. Functional experiments demonstrated that DKK1 silencing significantly impaired the proliferation, colony formation, migration, and invasion of MG-63 cells, supporting a pro-tumorigenic role for DKK1 under the tested *in vitro* conditions. DKK1 should therefore be regarded as a candidate secreted factor associated with the double-high phenotype rather than a downstream effector of a fatty acid metabolic axis.

Several limitations should be acknowledged. First, although single-cell sequencing provides high-resolution characterization of cellular states and communication networks, it lacks spatial information. Therefore, the physical proximity and spatial organization of IBSP+ malignant cells and SPARC-positive macrophages require further validation using spatial transcriptomics or multiplex imaging approaches. Second, functional validation in this study was primarily conducted *in vitro*, which cannot fully recapitulate the complexity of the bone tumor microenvironment. Future studies employing orthotopic mouse models and patient-derived xenografts will be necessary to evaluate the therapeutic potential of targeting DKK1 and lipid metabolic pathways *in vivo*. Finally, the cell–cell communication network was computationally inferred from ligand–receptor expression patterns does not directly demonstrate physical cellular interactions, ligand secretion, receptor activation, or causal signaling. These predicted interactions require further validation using spatial colocalization, protein-level assays, and functional coculture experiments.

In summary, our study links single-cell transcriptomic heterogeneity with clinical outcomes and functional validation, providing new insights into the role of metabolic reprogramming in osteosarcoma progression. We identify a previously unrecognized interaction between lipid-metabolism-active IBSP+ malignant cells and SPARC-positive macrophages, mediated in part through APOE and SPP1 signaling, and further establish DKK1 as a key downstream regulator associated with this aggressive microenvironment. These findings not only advance our understanding of osteosarcoma biology but also highlight potential opportunities for prognostic stratification and therapeutic intervention targeting metabolic and immune regulatory pathways.

## Data Availability

Publicly available datasets were analyzed in this study. The single-cell and bulk transcriptomic datasets analyzed during the current study are available in public repositories. Single-cell RNA sequencing datasets via the NCBI Gene Expression Omnibus (GEO) database under the accession numbers GSE152048 and GSE162454. Bulk RNA-sequencing datasets through the TARGET-OS project hosted on the Genomic Data Commons (GDC) Data Portal, as well as the GEO database under accession numbers GSE21257 and GSE33382. All original code and intermediate data dependencies generated during the analysis are available from the corresponding author upon reasonable request.
